# Hemodynamic evolution of heart failure from hypertension: lower stroke volume index and higher heart rate

**DOI:** 10.1093/ajh/hpag067

**Published:** 2026-07-03

**Authors:** Joseph L Izzo, Sambasiva R Srungavarapu, Chander P Khatri, Fnu Shalini, Peter J Osmond

**Affiliations:** Department of Medicine, Jacobs School of Medicine, University at Buffalo, Buffalo, NY, United States; Department of Medicine, Jacobs School of Medicine, University at Buffalo, Buffalo, NY, United States; Department of Medicine, Jacobs School of Medicine, University at Buffalo, Buffalo, NY, United States; Department of Medicine, Jacobs School of Medicine, University at Buffalo, Buffalo, NY, United States; Department of Medicine, Jacobs School of Medicine, University at Buffalo, Buffalo, NY, United States

**Keywords:** hypertension, hemodynamics, heart failure, heart rate, stroke volume, blood pressure

## Abstract

**Background:**

There is little information about systemic hemodynamic changes in the evolution of heart failure (HF) from hypertension.

**Methods:**

We compared 24-hour mean hemodynamic variables derived from ambulatory pulse wave analysis (Mobil-O-Graph) in at-risk patients with hypertension (Stage A/B HF) and patients with Stage C HF secondarily stratified by ejection fraction (EF: low <40% or high ≥40%).

**Results:**

Differences (mean ± SEM) between Stage A/B (n = 109) and Stage C HF (n = 52) were, respectively: stroke volume index (SVI) 36.8 vs. 31.9 mL/m^2^ (*P* < .001), heart rate (HR) 68.1 vs. 76.4 bpm (*P* < .001), and arterial stiffness (pulse pressure/SVI) 1.47 vs. 1.65 mmHg/mL/m^2^ (*P* < .05); cardiac index and total vascular resistance index did not differ between groups. Robust inverse correlations between SVI and HR were found in both Stage A/B and Stage C HF (*P* < .001); each 10 beat/min change in HR was accompanied by a reciprocal SVI change of 3 mL/m^2^; overall, SVI was 5%-10% lower in Stage C compared to Stage A/B HF. Hemodynamic profiles, especially high HR and low SVI, were quantitatively similar in Stage C HF patients with low or high EF. Low SVI (<35 mL/min/m^2^) conferred a specificity for Stage C HF of 71%; adding high HR (>75 bpm) increased specificity to 84%.

**Conclusions:**

Ambulatory hemodynamic monitoring is feasible in HF patients; low 24-hour SVI and high HR are highly correlated and together can differentiate Stage C from Stage A/B HF, irrespective of EF. Further study of hemodynamic profiles in HF staging and prognosis is warranted.

## Graphical abstract

**Graphical Abstract:**
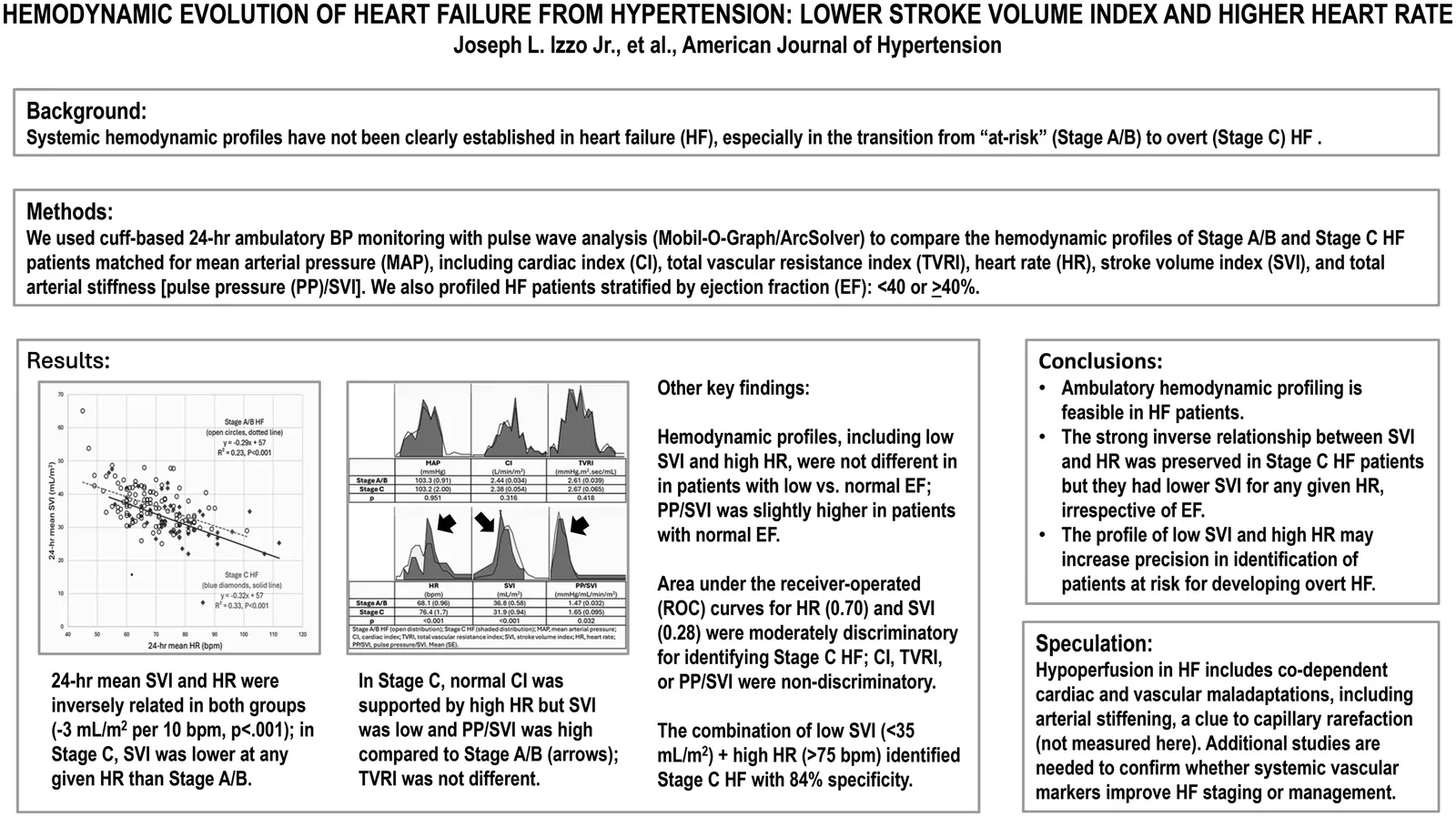

## Introduction

Hypertension (HTN) is primarily a hemodynamic syndrome that is the most prevalent antecedent of heart failure (HF).^1–3^ HF has been historically conceptualized as systemic hypoperfusion, where the heart cannot pump enough blood to meet the body’s demands. Hypoperfusion may not always include reduced cardiac contractility and systemic blood flow; emerging concepts suggest that concomitant macro- and micro-vascular abnormalities, including large artery stiffening, arteriolar thickening, abnormal microcirculatory pulsatility, and capillary rarefaction also play important roles.^4–7^ Another relatively universal feature of HF is myocardial stiffness with impaired ventricular relaxation, neither of which are closely related to cardiac ejection fraction (EF).^7^^,^^8^ All of these features are associated with adverse outcomes in HF patients.^7^^,^^9–12^

There is surprisingly little information regarding the hemodynamic evolution of HF from HTN. By convention, the 4 stages of HF are: Stage A, at-risk asymptomatic patients (including all patients with HTN); Stage B, at-risk asymptomatic patients with cardiac structural change or early signs of volume overload; Stage C: symptomatic patients with at least 1 episode of acute fluid overload; and Stage D, end-stage disease.^13^ Organ perfusion remains difficult to quantitate, so cardiac structure-function indicators such as ejection fraction (EF) or atrial volumes are commonly measured.^13^ To date, hemodynamic variables such as stroke volume index (SVI) and heart rate (HR) have not been included in HF staging criteria even though they portend worse outcomes in patients with established HF.^14–21^ Cuff-based 24-hour pulse wave analysis can be used to generate systemic hemodynamic profiles in ambulatory patients and we have previously identified a strong inverse relationship between HR and stroke volume^22^^,^^23^ but have not previously applied these methods in HF patients.

In this preliminary hypothesis-generating study, we have addressed 3 interrelated questions: (1) Is the reciprocal relationship between stroke volume and HR preserved in HF patients? (2) Are there different systemic hemodynamic profiles between Stage A/B and Stage C HF that could help in HF evaluation? and (3) Do hemodynamic profiles differ in patients with low vs. high EF?

## Methods

This investigation conformed to all principles of the Declaration of Helsinki (1996).

### Patient population

Our database included all HTN and HF patients referred for courtesy 24-hour ambulatory blood pressure monitoring (ABPM) and 26 participants in a clinical HTN trial (NCT05170061). The University at Buffalo Health Sciences IRB waived the need for a separate consent form for data acquisition but requested creation of a limited-access database and deidentification prior to analysis.

All patients with HTN had at least one office visit with systolic BP ≥140 mmHg and mean 24-hour ambulatory systolic BP ≥130 mmHg or were taking antihypertensive drugs. HF patients had at least one documented episode of fluid overload requiring hospital admission or acute ambulatory care with emergency parenteral diuretic given for new-onset dyspnea or edema and evidence of pulmonary or venous congestion, elevated plasma NT-proBNP, and at least 1 structure/function abnormality on echocardiogram (eg, increased atrial or ventricular volume). Etiology of HF was also considered, with special attention to current or antecedent HTN and ischemic heart disease (IHD, including coronary artery disease, prior MI, or history of coronary intervention). Patients with diabetes, ischemic heart disease, or atrial fibrillation were included in the analysis but no patients with Stage G4-5 CKD, untreated valvular heart disease, or other cardiomyopathies were referred for study.

We constructed 2 study cohorts: those with “pre-HF” (ie, either Stage A or B HF) served as a control group, while the comparison group had documented Stage C HF. We combined Stages A and B HF because it is not possible to accurately define Stage B HF due to the fact that asymptomatic at-risk individuals (largely those with a prior or current diagnosis of HTN) are not routinely studied with echocardiography or other imaging studies and thus may have abnormalities consistent with Stage B criteria that are undetected.

### Ambulatory hemodynamic monitoring

We used a Mobil-O-Graph (IEM, Stolberg, Germany) with pulse wave analysis software (ARCSolver, Austrian Institute of Technology), with readings taken every 20 minutes for 24 hours. Quality criteria required a minimum of 36 valid 10-second readings, including systolic BP, diastolic BP, mean arterial pressure [MAP], and pulse pressure [PP]. Hemodynamic variables were calculated according to the standard formula: MAP = cardiac output (CO) × total vascular resistance (TVR); CO was decomposed into its primary components, heart rate (HR) and stroke volume. All but MAP and HR were normalized by body surface area and reported as respective index (I) values. Total arterial stiffness was estimated as PP/SVI.

### Data analysis

TVRI and PP/SVI, and to a lesser extent CI and SVI, are all BP-related so valid comparison of hemodynamic profiles between Stage A/B and Stage C HF cohorts required BP adjustment. To do this and at the same time to maximize the number of subjects included in the study, we first calculated the mean MAP for the smaller Stage C cohort and then included as many Stage A/B-HF patients as possible to achieve group mean MAP values within 0.1 mmHg.

IBM SPSS Statistics (Ver 30) was used for statistical and graphic analyses. Differences in demographics were evaluated by Student *t*-tests. The impacts of demographic imbalances on final results were assessed by sensitivity analyses; we stratified by any unbalanced demographic characteristic (eg, men, whites, and non-diabetics) and compared the hemodynamic profile of the majority subgroup to that of the full cohort. Relationships between SVI and HR in Stage A/B and Stage C patients were studied using standard Pearson regressions; the respective slopes from the 2 regression equations (SVI[*y*] − SVI[*y* mean])/([HR[*x*] − HR[*x* mean]) were compared by Student *t*-test, which was also used in the primary hemodynamic comparisons between Stage A/B and Stage C patients and in the secondary comparisons between patients with low (<40%) and high EF (≥40%). Predictive values for Stage C HF for the major hemodynamic variables were calculated using the area under the curve (AUC) for the respective receiver-operated characteristic (ROC) curves; specificity and sensitivity were also calculated for candidate predictor variables.

## Results

### Patients

Table 1 shows the basic characteristics of the primary comparison (Stage A/B vs Stage C HF) and the secondary comparison (HF patients with EF < 40 vs EF ≥ 40). Fifty-one of 52 Stage C HF patients (98%) had a history of HTN and 14 (27%) had proven ischemic heart disease. Medications at the time of 24-hour ambulatory monitoring are listed for each group for informational purposes but were not analyzed statistically. During group mean MAP matching, we eliminated 23 of the total available cohort of 132 Stage A/B patients; results from a secondary analysis of MAP-unmatched patients are presented in Table S1; we found no appreciable differences between the total Stage A/B cohort (n = 132) and the MAP-matched Stage A/B cohort (n = 109) with respect to mean HR, CI, TVRI, or SVI but arterial stiffness (PP/SVI), which is most directly BP-dependent, was higher in the unmatched Stage A/B cohort because the full Stage A/B cohort included a few individuals with very high systolic BP values.

**Table 1:** Patient characteristics.

	Stage A/B HF	Stage C HF				
		All	*P* Stage A/B vs Stage C	EF ≥ 40	EF < 40	*P* EF ≥ 40 vs EF < 40
N	109	52	161	23	29	52
**Demographics and blood pressures**						
**Age (yrs)**	62 (1.4)	61 (1.7)	.722	57 (1.9)	66 (2.6)	.007
**Weight (kg)**	85 (1.9)	95 (3.4)	.007	97 (4.6)	93 (4.9)	.531
**Gender (n, % male)**	54 (49%)	36 (69%)	.019	13 (56%)	23 (79%)	.080
**Race (n, % Black)**	27 (24%)	34 (65%)	<.001	17 (74%)	17 (74%)	.793
**IHD (n[%])**	N/A	14 (27%)	–	5 (21%)	9 (31%)	.539
**Diabetes (n[%])**	14 (13%)	21 (40%)	<.001	7 (30%)	14 (48%)	.190
**Prior/current HTN**	109 (100%)	51 (98%)	.320	23 (100%)	38 (97%)	.378
**24-SBP (mmHg)**	132 (1.2)	130 (2.5)	.420	137 (3.7)	125 (3.3)	.029
**24-DBP (mmHg)**	79 (.86)	80 (1.8)	.466	80 (2.6)	80 (2.7)	.932
**24-PP (mmHg)**	53 (1.0)	50 (1.7)	.079	56 (2.8)	45 (1.8)	.001
**Medications (n, %)**						
**Diuretic**	39 (38%)	45 (86%)	<.001	22 (96%)	23 (79%)	.090
**RASi**	58 (57%)	40 (76%)	.014	17 (74%)	23 (79%)	.654
**CCB**	39 (38%)	12 (23%)	.059	7 (30%)	5 (17%)	.271
**Beta blocker**	43 (42%)	10 (82%)	<.001	20 (87%)	23 (79%)	.479
**H/N**	2 (2%)	7 (13%)	.004	5 (22%)	2 (7%)	.124
**SGLT2i**	0 (0%)	7 (13%)	<.001	6 (26%)	1 (3%)	.017
**Other**	8 (8%)	4 (8%)	.976	3 (13%)	1 (3%)	.205
**# CV Meds/patient**	1.9 (0.13)	3.0 (0.15)	<.001	3.5 (0.21)	2.7 (0.19)	.008

IHD, ischemic heart disease; HTN, hypertension; HF, heart failure; 24, 24-hour ambulatory mean; SBP, systolic BP; DBP, diastolic BP; PP, pulse pressure; EF, ejection fraction; RASi, renin-angiotensin inhibitor (ACE inhibitor, ARB, or valsartan-sacubitril); CCB, calcium channel blocker; H/N hydralazine/nitrate; SGLT2i, sodium-glucose linked transporter inhibitor; Other (clonidine, alpha blocker). Mean (SE) unless otherwise specified.

Sensitivity analyses for unbalanced demographic characteristics between Stage A/B and Stage C patients are presented in Tables S2-S5. Stage C patients were heavier than Stage A/B patients (95 vs 85 kg, *P* < .05) but that is why CO, TVR, and SV were represented as index values. In the MAP-matched Stage A/B cohort compared to the Stage C cohort, there were more men than women (69% vs 49%, *P* < .05), more whites than Blacks (65% vs 24%, *P* < .001) and more non-diabetics than diabetics (13% vs 40%, *P* < .001). In Stage C HF patients, diabetes mellitus was present in 30% of patients with EF ≥40% and 48% of patients with EF < 40% (pNS), while IHD was present in 21% with EF < 40% and 31% with EF ≥ 40 (pNS). None of the demographic imbalances in gender, race, or diabetes prevalence between the MAP-matched Stage A/B cohort and the Stage C HF cohorts affected the primary hemodynamic comparisons.

**Figure 1:**
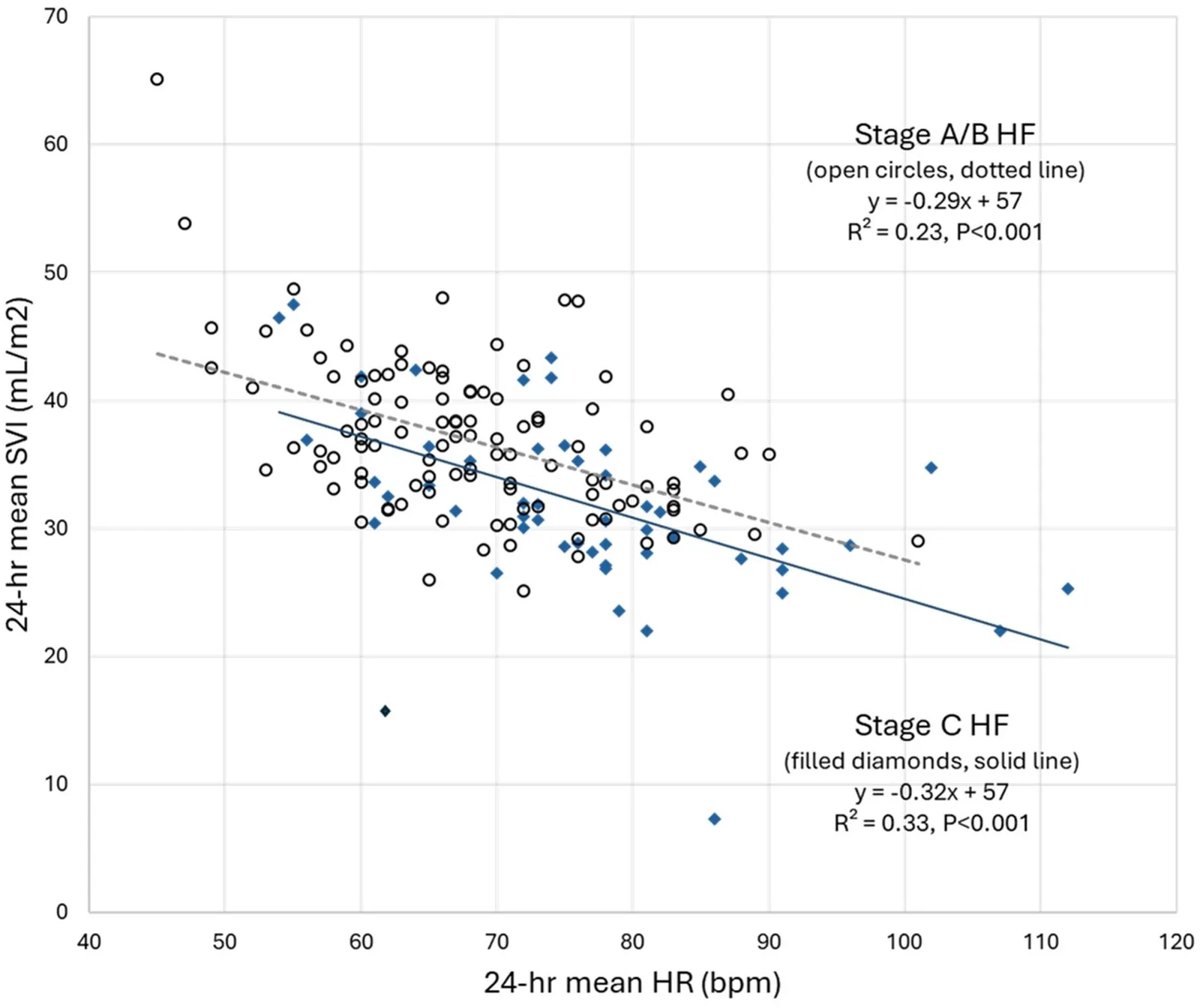
Counter-regulation of SVI and HR. In both groups, there were similar robust inverse relationships between SVI and HR but in stage C HF, there was a parallel downward shift in the curve compared to Stage A/B HF.

Figure 1 depicts the inverse correlations between 24-hour SVI and 24-hour HR in Stage A/B and Stage C HF populations (*r*^2^ 0.23 and 0.33, respectively, *P* < .001 each). The slopes of the SVI-HR regression equations in the Stage A/B and Stage C cohorts were nearly parallel (0.29 vs. 0.33 mL/m^2^/bpm, respectively, *P* = .98); thus, in both groups, each change in HR of 10 bpm was associated with a reciprocal change in SVI of about 3 mL/m^2^.

**Figure 2:**
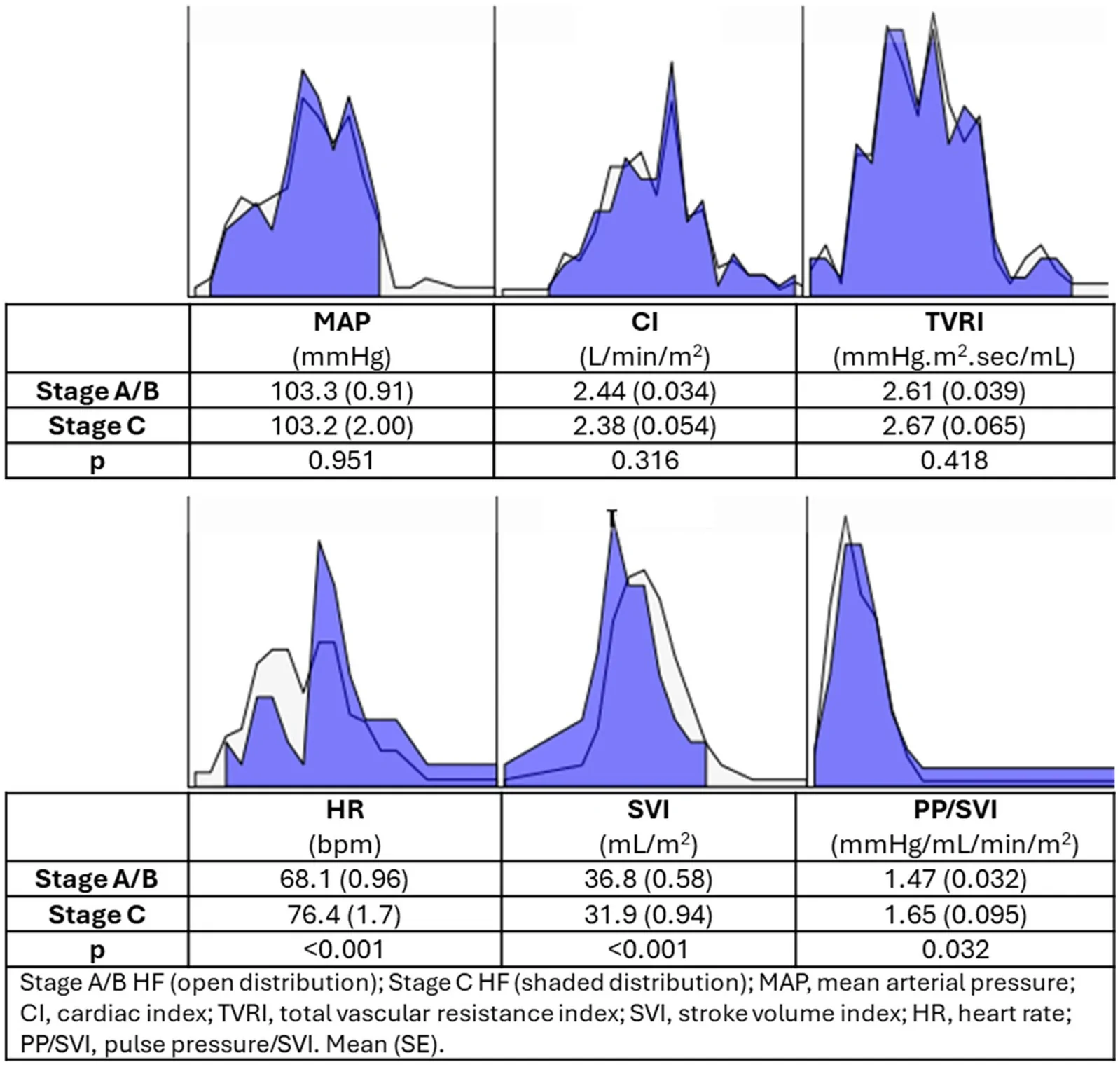
Hemodynamic comparison of Stage A/B and stage C HF. In subjects matched for MAP, there were no differences between groups in CI or TVRI but SVI was lower and HR and arterial stiffness (PP/SVI) were higher in stage C HF than in Stage A/B HF patients.

Figure 2 demonstrates comparative values for the 6 core hemodynamic indicators (MAP, CI, TVRI, HR, SVI, and PP/SVI) in Stage A/B and Stage C HF populations. Between groups, there were no differences in the primary components of MAP (CI and TVRI) but HR was significantly higher (76 vs 68 bpm, *P* < .001) and SVI was significantly lower (31.9 vs 36.8 mL/m^2^, *P* < .001) in Stage C compared to Stage A/B HF. Whole-body arterial stiffness (PP/SVI) was also higher in Stage C HF patients (*P* < .05).

**Figure 3:**
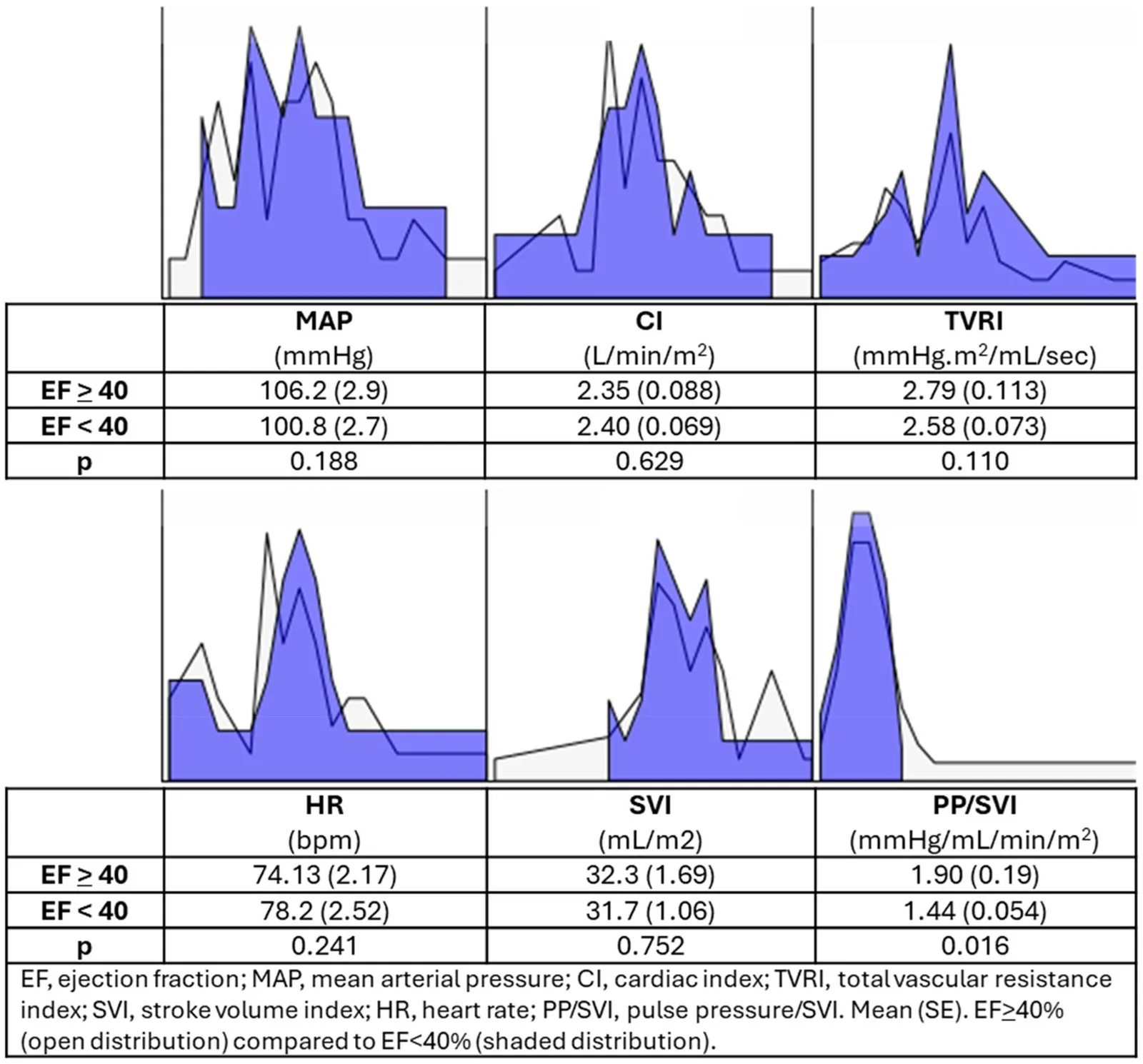
Hemodynamic comparison of stage C HF patients stratified by EF. There were no hemodynamic differences between those with low and high EF except for arterial stiffness (PP/SVI), which was higher in patients with EF ≥ 40%.

Figure 3 demonstrates the same core hemodynamic indicators as in Figure 2 in HF patients stratified by EF (<40% compared to ≥40%). There were no differences in MAP, CI, TVRI, HR, or SVI between low and high EF subgroups but PP/SVI was higher in those with EF ≥ 40% (*P* = .016).

**Figure 4:**
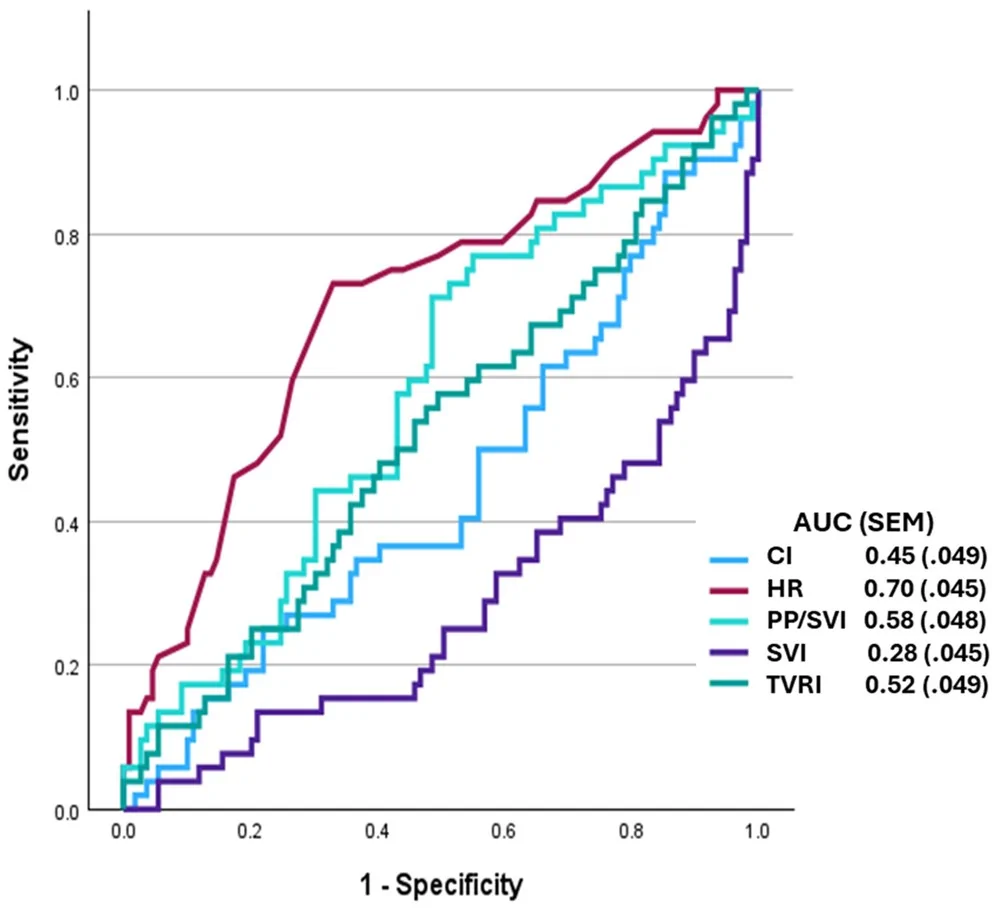
Receiver-operated characteristic curves for individual hemodynamic variables in the discrimination of stage C from stage a/B HF. Abbreviations as in Figures 2 and 3. AUC is area under the curve (SEM).

Figure 4 demonstrates the respective ROC curves and corresponding area under the curve (AUC) for core hemodynamic indicators. Higher HR and lower SVI were similar in their discriminant powers to detect Stage C HF (0.70 ± .045 and 0.28 ± .045, respectively). For comparison, the AUC of 1/SVI to identify Stage C HF was 0.72 ± .045. AUC values (SEM) for other variables were not discriminant: PP/SVI 0.58 (.048), CI 0.45 (.049), and TVRI 0.52 (.049). Low SVI, defined in other studies as <35 mL/m^2^, had sensitivity and specificity for Stage C HF of 57% and 71%, respectively, while high HR, defined arbitrarily as >75 bpm, had sensitivity and specificity for Stage C HF of 56% and 74%, respectively. When low SVI (<35 mL/m^2^) and high HR (>75 bpm) were combined, sensitivity and specificity for Stage C HF were 48% and 84%, respectively. If the SVI cutoff was lowered to <32 mL/m^2^, sensitivity and specificity for Stage C HF were 59% and 75%; if the HR cutoff was lowered to >72 bpm, sensitivity and specificity were 63% and 68%, respectively, while sensitivity and specificity for the combined cutoff values of SVI < 32 mL/m^2^ and HR > 72 bpm were 46% and 89%, respectively.

## Discussion

With respect to our experimental questions, we found that the strong negative correlation between 24-hour ambulatory SVI and 24-hour ambulatory HR previously identified in normal and hypertensive individuals^23^ was preserved in Stage C HF. Stage C HF was characterized by low SVI and high HR, irrespective of EF, and sensitivity analyses suggested that this hemodynamic profile was not affected by race, gender, diabetes status, or SGLT2 inhibitor use. With respect to ROC analysis, the AUC values to identify Stage C HF for low SVI (<35 mL/m^2^) and high HR (>75 bpm) demonstrated only modest predictive value, but when combined, low SVI and high HR identified Stage C HF with a specificity of 84%, a reasonably high predictive value. In addition to history of HTN, a practical clinical clue to the Stage C HF hemodynamic profile was high arterial stiffness.

Hypertension (HTN) is at the top of the lists of causes and comorbidities of HF.^1–3^^,^^7^ Not only is HTN the most prevalent HF risk factor, it can also be viewed as part of an HTN-HF continuum that has several shared pathogenetic features including increased neurohumoral activity, heart rate (HR), and arterial stiffness but we did not find higher TVRI in HF patients.^24–28^ Another important similarity between HTN and HF is that both conditions respond to many of the same drug classes (especially diuretics, renin-angiotensin-aldosterone blockers, beta blockers, and SGLT-2 inhibitors), which lower BP, improve HF symptoms, and reduce cardiovascular morbidity and mortality.^29–32^ Even though an expert panel has proposed a relatively concrete definition of HF as a clinical syndrome characterized by cardiac structure-function abnormalities,^13^ this approach has tended to deflect attention from the HTN-HF continuum and original concept of HF as systemic hypoperfusion.

In contrast to most current structure-function indicators, it is possible that low SVI and high HR may be indirect markers of tissue hypoperfusion, which can theoretically occur even if systemic blood flow is not markedly reduced. We did not directly assess microcirculatory function in this study, so the following discussion could be considered speculation. However, powerful emerging concepts strongly suggest that HF is best appreciated as combined cardiac and vascular dysfunction leading to systemic hypoperfusion. Perfusion is a complex phenomenon that may include reduced blood flow or pulse volume but also can derive from vascular factors including abnormal pulse wave transmission (eg, arterial stiffness), vascular occlusions, selective neurogenic control of regional impedance with regional blood flow redistribution, altered macrocirculatory and microcirculatory pulsatility, and microvascular structure-function abnormalities including variation in capillary density (or surface area) and “dwell time.” Capillary rarefaction, defined as either functional or anatomic capillary loss, is of particular interest because it may be a “final common pathway” in the pathogenesis of interlinked cardiovascular and renal disorders, including essential hypertension, diabetes, chronic kidney disease, and HF.^4–7^^,^^33–40^ Myocardial capillary rarefaction is associated with abnormal cardiac oxygen consumption, low exercise tolerance, and impaired ventricular relaxation in HF patients.^36–39^ With capillary rarefaction, the average distance of a parenchymal cell from the nearest functional capillary increases, along with diffusion time for oxygen and nutrients. Reduced capillary cross-sectional area promotes higher capillary blood flow velocity but also reduces the time available for diffusion of oxygen and substrate within each pulse volume (capillary “dwell time”). High HR further exacerbates this defect. Capillary rarefaction is strongly correlated with large artery stiffness,^4^^,^^5^ so the latter serves as a clinical clue to capillary rarefaction.

The pathogenesis of the strong inverse relationship between SVI and HR^23^^,^^41^^,^^42^ may represent opposing influences on the sympathoadrenal system and mutual rather than linear causality. It is well established that sympathetic nervous activity is increased in HF^24^^,^^43^ and sympathoexcitation directly raises HR,^44^ which reduces ventricular filling time and SVI. Thus, it can be argued that high HR is a primary cause of low SVI. On the other hand, a primary reduction in SVI can cause sympathoexcitation via renal and skeletal muscle metaboreceptors, if low SVI causes microcirculatory hypoperfusion with increased anaerobic metabolism and reduced tissue oxygen content.^44^ Pharmacologic lowering of HR promotes an increase in SVI but does not shift the SVI-HR curve downward as observed in HF patients.^23^ In contrast, the lower SVI in Stage C HF patients could also be considered the primary hemodynamic abnormality. Whichever is primary, both high HR and low SVI have negative prognostic value in patients with established Stage C HF, irrespective of EF classification or presence of atrial fibrillation.^15^^,^^19–21^ Our data also confirm that high total arterial stiffness (PP/SVI) is common in Stage C HF patients, particularly in those with EF ≥ 40%. Increased arterial stiffness also has a potentially negative impact on ventricular contractility and relaxation and signals poorer HF outcomes.^45^^,^^46^ What remains to be proven is if low SVI or high HR, alone or together, can predict the evolution of Stage C from Stage A/B HF. Perhaps high arterial stiffness can be used as a clinical clue that could prompt hemodynamic profiling to detect combined cardiac and vascular dysfunction in at-risk HF patients.

There are other indirect implications of present findings. A prime example is the highly questionable practice of labeling all patients with hypertension as having HF (Stage A) when the vast majority of this population will never develop HF. Based on today’s definitions, the prevalence of HTN in US adults is about 50% (∼120 million) and the lifetime risk of HTN exceeds 90%; in contrast, the prevalence of overt HF is <3% (∼7 million) and the lifetime risk of HF is <25%.^47–50^ It is clear from these trends that more specific HF risk stratification indicators are needed. Another practice that can be challenged is the over-reliance on EF in HF care. EF has proven prognostic significance when very low (eg, 77% excess morbidity when <15%) but the practical cutoff value for low EF and the general use of EF in HF management continue to be actively debated.^14^^,^^17^^,^^20^^,^^21^^,^^51^^,^^52^ There are several shortcomings in the general understanding and clinical use of EF. First, EF is heavily dependent on its denominator (EF = stroke volume/end-diastolic volume as %) and in contrast to HR and SVI, is not directly related to systemic blood flow.^14^^,^^53^ Second, EF is quite variable within and between individuals, due in part to the inverse relationship of SVI and HR. In one study, pacemaker-induced changes in SVI and EF changes were correlated, with each 10 beat/min change in HR associated with a reduction of EF of about 4.4%, which is similar to what we have observed for SVI.^23^^,^^41^ This further suggests that EF should probably be reported as both absolute and HR-normalized values, which is not current practice, or perhaps combined with HR to improve diagnostic accuracy. Other cardiovascular risk factors have at least comparable predictive value, including high HR (each 5 beat/min increase augments CVD risk by about 12%), low SVI (a 69% increment for each SD in the population), and high PP/SVI (about 63% greater MACE rates).^54–56^

Like all retrospective, observational, hypothesis-generating investigations, our study has weaknesses and limitations and accordingly, interpretation and extrapolation must be appropriately tempered. Our data represent a small convenience sample but there was no attempt to select the population that was available. Our goal was to provide an initial comparison of evolving hemodynamic profiles (CI, HR, SVI, and TVRI, and PP/SVI) in Stage C HF compared to Stage A/B “pre-HF” with the assumption that our cross-sectional study could yield information on disease progression similar to a prospective follow-up investigation. Our results were statistically significant, consistent with other studies. Although demographic characteristics such as age, race, gender, body size, and diabetes status influence cardiovascular disease, we did not find that these factors influenced our overall interpretation or conclusions, which we believe are potentially clinically meaningful.

## Conclusions

We conclude that ambulatory hemodynamic profiling is feasible and potentially useful in HF care. The strong inverse relationship between SVI and HR is preserved in Stage C HF patients, who also had lower SVI at any given HR, irrespective of EF. The combination of low SVI (<35 mL/m^2^) and high HR (>75 bpm) conferred a specificity for identifying Stage C HF of 84%. We suggest that further confirmatory studies are warranted.

## Supplementary Material

hpag067_Supplementary_Data

## Data Availability

Original deidentified data can be made available on request.
