# Supplementary material for: Hemodynamic evolution of heart failure from hypertension: lower stroke volume index and higher heart rate
Source: Am J Hypertens. 2026 Jul 3;39(10):1222–30. doi: 10.1093/ajh/hpag067 (PMC13579055; doi:10.1093/ajh/hpag067)
Supplement: Supplementary file 1 [file hpag067_supplementary_data.docx]

| Supplemental Table S1: Hemodynamic profiles by HF Stage in patients not matched for MAP | | | |
| --- | --- | --- | --- |
| Variable | All Stage A/B | All Stage C | p |
| n | 132 | 52 |  |
| Systolic BP | 137.6 (1.46) | 130.3 (2.55) | 0.011 |
| Diastolic BP | 82.3 (1.00) | 80.2 (1.85) | 0.291 |
| Pulse pressure | 55.3 (1.01) | 50.1 (1.73) | 0.008 |
| MAP | 107.6 (1.13) | 103.2 (2.01) | 0.049 |
| CI | 2.46 (0.033) | 2.39 (0.054) | 0.242 |
| HR | 68.5 (0.92) | 76.4 (1.71) | <0.001 |
| SVI | 36.8 (0.52) | 32.0 (0.95) | <0.001 |
| TVRI | 2.72 (0.042) | 2.68 (0.066) | 0.623 |
| PP/SVI | 1.53 (0.031) | 1.66 (0.096) | 0.106 |
| Data are Mean (SE). Abbreviations as in Table 1. See also Figure 2. | | | |

| Supplemental Table S2: Hemodynamic profiles in males by HF Stages | | | |
| --- | --- | --- | --- |
| Variable | Stage A/B | Stage C | p |
| n | 82 | 18 |  |
| Systolic BP | 132.4 (1.38) | 130.5 (3.23) | 0.517 |
| Diastolic BP | 80.8 (1.14) | 81.8 (2.29) | 0.658 |
| Pulse pressure | 51.6 (1.17) | 48.7 (2.13) | 0.191 |
| MAP | 104.5 (1.11) | 104.2 (2.53) | 0.906 |
| CI | 2.36 (0.045) | 2.36 (0.069) | 1.000 |
| HR | 67.8 (1.37) | 76.9 (2.13) | <0.001 |
| SVI | 35.8 (0.89) | 31.3 (1.20) | 0.003 |
| TVRI | 2.74 (0.053) | 2.76 (0.085) | 0.886 |
| PP/SVI | 1.48 (0.046) | 1.55 (0.60) | 0.134 |
| Data are Mean (SE). Abbreviations as in Table 1. See also Figure 2. | | | |

| Supplemental Table S3: Hemodynamic profiles in whites by HF Stages | | | |
| --- | --- | --- | --- |
| Variable | Stage A/B | Stage C | p |
| n | 82 | 18 |  |
| Systolic BP | 131.2 (1.43) | 125.9 (3.23) | 0.128 |
| Diastolic BP | 77.7 (1.02) | 75.7 (2.98) | 0.422 |
| Pulse pressure | 53.5 (1.18) | 50.1 (2.02) | 0.210 |
| MAP | 102.2 (1.07) | 98.8 (2.90) | 0.200 |
| CI | 2.43 (0.038) | 2.35 (0.093) | 0.366 |
| HR | 67.5 (1.08) | 77.4 (3.76) | <0.001 |
| SVI | 37.0 (0.65) | 31.4 (2.04) | 0.001 |
| TVRI | 2.61 (0.045) | 2.62 (0.10) | 0.939 |
| PP/SVI | 1.47 (0.036) | 1.80 (0.235) | 0.015 |
| Data are Mean (SE). Abbreviations as in Table 1. See also Figure 2. | | | |

| Supplemental Table S4: Hemodynamic profiles in non-diabetics by HF Stages | | | |
| --- | --- | --- | --- |
| Variable | Stage A/B | Stage C | p |
| n | 95 | 31 |  |
| Systolic BP | 131.6 (1.24) | 130.5 (3.72) | 0.708 |
| Diastolic BP | 78.5 (0.94) | 80.0 (2.56) | 0.510 |
| Pulse pressure | 53.2 (1.07) | 50.5 (2.46) | 0.256 |
| MAP | 102.8 (0.94) | 103.1 (2.88) | 0.884 |
| CI | 2.46 (0.039) | 2.35 (0.069) | 0.191 |
| HR | 67.9 (0.96) | 73.5 (1.93) | 0.007 |
| SVI | 37.0 (0.61) | 32.8 (1.39) | 0.002 |
| TVRI | 2.60 (0.044) | 2.71 (0.094) | 0.257 |
| PP/SVI | 1.46 (0.034) | 1.66 (0.151) | 0.054 |
| Data are Mean (SE). Abbreviations as in Table 1. See also Figure 2. | | | |

| Supplemental Table S5: Hemodynamic profiles of patients not taking SGLT2 inhibitors by HF stages | | | |
| --- | --- | --- | --- |
| Variable | Stage A/B | Stage C | p |
| n | 109 | 45 |  |
| Systolic BP | 132.3 (1.21) | 130.2 (2.89) | 0.420 |
| Diastolic BP | 78.9 (0.86) | 80.1 (2.06) | 0.532 |
| Pulse pressure | 53.4 (1.00) | 50.2 (1.97) | 0.104 |
| MAP | 100.4 (1.13) | 103.1 (2.26) | 0.889 |
| CI | 2.45 (0.035) | 2.38 (0.054) | 0.280 |
| HR | 68.2 (0.96) | 76.8 (1.89) | <0.001 |
| SVI | 36.9 (0.59) | 31.8 (0.84) | <0.001 |
| TVRI | 2.62 (0.040) | 2.67 (0.070) | 0.478 |
| PP/SVI | 1.60 (0.061) | 1.51 (0.029) | 0.066 |
| Data are Mean (SE). Abbreviations as in Table 1. See also Figure 2. | | | |
